# Mathematical Modelling of Metabolic Regulation in Aging

**DOI:** 10.3390/metabo5020232

**Published:** 2015-04-27

**Authors:** Mark T. Mc Auley, Kathleen M. Mooney, Peter J. Angell, Stephen J. Wilkinson

**Affiliations:** 1 Faculty of Science & Engineering, University of Chester, Thornton Science Park, CH2 4NU, UK; E-Mail: s.j.wilkinson@chester.ac.uk; 2 Faculty of Health and Social Care, Edge Hill University, Ormskirk, Lancashire, L39 4QP, UK; E-Mail: kathleen.mooney@edgehill.ac.uk; 3 School of Health Sciences, Liverpool Hope University, Taggart Avenue, Liverpool, L16 9JD, UK; E-Mail: angellp@hope.ac.uk

**Keywords:** aging, computational modelling, mammalian target of rapamycin, systems biology, simulation, sirtuins, SIRT1, regulatory network

## Abstract

The underlying cellular mechanisms that characterize aging are complex and multifaceted. However, it is emerging that aging could be regulated by two distinct metabolic hubs. These hubs are the pathway defined by the mammalian target of rapamycin (mTOR) and that defined by the NAD+-dependent deacetylase enzyme, SIRT1. Recent experimental evidence suggests that there is crosstalk between these two important pathways; however, the mechanisms underpinning their interaction(s) remains poorly understood. In this review, we propose using computational modelling in tandem with experimentation to delineate the mechanism(s). We briefly discuss the main modelling frameworks that could be used to disentangle this relationship and present a reduced reaction pathway that could be modelled. We conclude by outlining the limitations of computational modelling and by discussing opportunities for future progress in this area.

## 1. Introduction

One of the great challenges in biology is to understand the underlying mechanisms of aging. Aging is recognized as a deleterious process that occurs during the lifespan of an organism, gradually rendering it increasingly vulnerable to mortality [1]. Aging is characterized by its complexity and that it affects all aspects of biology, from molecular processes to the whole organism. Cells are the essential building blocks of the human body; therefore, it is logical that mammalian cellular decline underpins the aging process. Over the years, several cellular mechanisms have been suggested as the underlying cause of aging. For example, cellular senescence, a phenomenon that occurs when normal differentiating cells stop dividing, was widely regarded as a viable model of aging [2,3]. However, it became apparent that senescence can accompany a diverse range of other cellular events. For instance, Harley *et al.* (1990) revealed that telomere shortening is associated with cellular senescence [4], and in certain cell lines, cells can even cause senescence (reviewed in [5]). In addition, mitochondria have been long recognized as a source of cellular damage, due to the deleterious consequences of reactive oxidative species (ROS) escaping from the mitochondrial electron transport chain during cellular respiration [6,7,8]. ROS can compromise the integrity of the cell by damaging lipids, proteins and nuclear/mitochondrial DNA [9]. For this reason, the production of ROS has been associated with telomere dysfunction [10] and cellular senescence, demonstrating the intertwined nature of aging [11,12]. Crosstalk among a variety of other cellular activities has also been implicated in how aging unfolds [13,14,15]. Thus, a general consensus has evolved that mammalian aging is not dictated by a single cellular event operating in isolation; it is the result of the interplay of a diverse range of biological processes operating over a wide-range of spatial and temporal scales. As a result of these findings, it has been recognized that in order to gain a more complete understanding of the mechanics of aging, integration of multiple biological pathways need to be considered [16,17]. For example, Passos *et al.* (2010) used *in silico* interactome analysis and live cell microscopy to show the presence of a dynamic feedback loop between p21 and cellular ROS levels [11].

**Figure 1:**
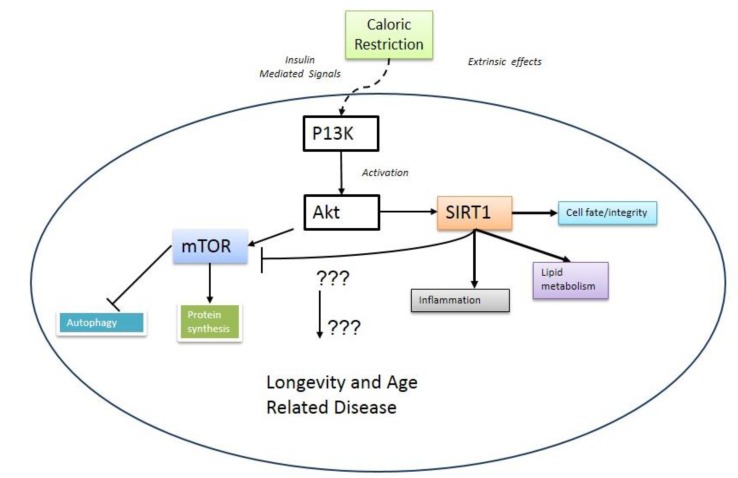
A coarse-grained overview of the metabolic pathways regulated by mTOR and SIRT1. The figure also shows SIRT1 inhibiting mTOR, as suggested by Ghosh *et al.* (2010).

Regardless of the approach that is adopted, a better understanding of aging is especially important due to an increase in age-related pathologies, such as cardiovascular disease (CVD) [18]. The aim of the systems biology paradigm is to provide an integrated interpretation and understanding of fundamental biological processes from the molecular through to the physiological [19,20,21,22,23]. Central to the systems biology approach is computational modelling, which involves using mathematics to quantitatively represent the dynamics of biological systems. Recently, computational models have been utilized to investigate the dynamics of aging, making it possible to intuitively represent the detailed networks of genes, proteins, metabolites and biochemical reactions that characterize this complex phenomenon [24,25,26,27]. A worthwhile resource for sourcing aging data at these different biological levels is the recently developed Digital Ageing Atlas (DAA) [28]. This is a freely accessible online database that has been designed specifically for archiving age-related data at various levels [28]. Within the dense biological circuitry that underpins aging, several important metabolic hubs are suggested to regulate longevity and health span. These include pathways defined by the mammalian target of rapamycin (mTOR) [29] and that defined by the NAD+-dependent deacetylase enzyme, SIRT1 [30]. mTOR and SIRT1 are involved in coordinating the availability of nutrients and energy to a wide range of cellular functions. Figure 1 presents a coarse-grained network diagram of these pathways, their interaction as proposed by Ghosh *et al*. (2010) and the cellular processes they impact [31]. It is possible that the intensity of the metabolic current passing through Figure 1 is modulated by caloric restriction (CR), a dietary regime that involves reducing nutrient intake without causing malnutrition [32,33]. This dietary regime has been shown to extend lifespan in a diverse range of organisms, and it is generally accepted that the effects of CR are at least partly mediated via these key metabolic pathways [34,35]. However, it must be stressed at this point that this is very much an open question, and it remains to be fully established if there is a link between CR and SIRT1 (recently reviewed in [36]). Despite this uncertainty, the relationship(s) between the mTOR and SIRT1 pathways have been the subject of intense scrutiny due to the potential they may offer for extending health span via pharmacological interventions that could replicate the effects of CR [37,38]. However, it remains unclear how their interplay might contribute to longevity. In this article, we explore mTOR and SIRT1 as metabolic sensors and the major pathways that they regulate. Experimental investigations that have examined their crosstalk will be discussed and a case presented for computationally modelling their interplay. A framework that could be used to do this will be outlined, and we will also define the challenges and opportunities that could influence how computational modelling is used in the future to investigate aging.

## 2. mTOR and Aging

mTOR is a serine/threonine protein kinase of the phosphatidylinositol-3-OH kinase (PI(3)K)-related family [39]. It acts as a key metabolic sensor in a wide range of biological effects, both at a cellular and organism level [40]. This ability to act as a regulator causes it to respond to an array of both intrinsic and extrinsic cellular signals. These signals include genotoxic stress, oxygen levels and hormone/nutrient levels [41,42,43]. mTOR forms the catalytic subunit of two discrete signaling complexes, known as mTOR complexes 1 and 2 (mTORC1 and mTORC2). In response to intracellular and extracellular cues, the mTOR pathway impacts cell growth and proliferation by stimulating anabolic processes, including biosynthesis of proteins, lipids and organelles, and by restricting catabolic processes, such as autophagy [44]. As a result, mTOR has been recognized as a key metabolic hub that can influence cell growth and survival [45]. Thus, it is logical to assume that mTOR could be a master metabolic controller with the potential to regulate both aging and the pathologies of old age. It is thus not surprising that recent efforts have focused on applying inhibitors of this pathway to the study of healthy aging [46]. For instance, evidence from mouse models suggests that inhibition of mTOR rescues cognitive deterioration, which is a clinical manifestation of Alzheimer’s disease [47,48,49]. Moreover, sporadic mutation or dysregulation of protein kinase B and the tuberous sclerosis complex, which are upstream components of the mTOR pathway, are frequently altered in cancer [50,51,52]. Another function of mTOR that has been associated with cancer development is tumour angiogenesis [53]. The mTOR kinase inhibitor rapamycin, also known as sirolimus, is an antifungal agent produced by the bacteria *Streptomyces hygroscopicus* and has long been touted as a therapeutic target of cancer [54,55]. For example, analogues of rapamycin have historically been used as chemotherapeutic agents against solid tumour types [56], including breast cancer [57,58]. Rapamycin has anti-angiogenesis effects, which are suggested to be mediated by lowering vascular endothelial growth factor (VEGF), a downstream product of the mTOR1 complex [59]. It could be very tentatively suggested, therefore, that over-activated mTOR promotes cancer growth by augmenting the supply of VEGF.

Rapamycin fed late in life has also been show to extend lifespan in genetically heterogeneous mice [60]. However, an alternative, more recent study has shown that, although rapamycin can extend lifespan in mice, it did not improve healthy aging [61]. Rapamycin, however, had similar effects on young animals, indicating that the effects of rapamycin were not due to a modulation of aging, but rather related to aging-independent drug effects. The life-extending effects of CR could, in part, be due to inhibition of mTOR, as suggested by the findings in *Drosophila* [62] and yeast [63]. CR has been shown to activate 4E-BP1 in *Drosophila*, resulting in increased translation of macromolecules involved in the mitochondrial electron transport chain and associated lifespan extension [64]. This increased functionality could be due to a reduction in ROS damage, as it has been suggested that inhibition of mTORC1 by rapamycin lowers mitochondrial membrane potential, oxygen consumption and cellular ATP levels [65,66], and it has been found that rapamycin inhibition can dramatically alter mitochondrial phosphoproteome [65]. It has been observed that mitochondrial DNA copy number, as well as the expression of several genes encoding proteins involved in oxidative metabolism are reduced by rapamycin and increased by mutations that activate mTORC1 signaling [66].

## 3. Metabolic Crosstalk between mTOR and SIRT1

Silent information regulator proteins, otherwise known as sirtuins (SIRTUINS), are an evolutionarily group of enzymes that operate as NAD+-dependent deacetylases [67]. There are seven known mammalian sirtuins that are localized to various parts of the cell [68]. Sirtuins take part in a diverse range of cellular activities, from nutrient sensing [68] to DNA damage/repair [69]. The response of sirtuins to environmental factors, as well as their potential role in aging is unclear. What is known is that SIRT1, the most extensively studied of the sirtuins, regulates various metabolic processes, which enable the cell to adapt to nutrient perturbations. In response to fasting, for example, SIRT1 modulates gluconeogenesis in the liver [70]. Moreover, SIRT1 has various other functions, including the regulation of fat mobilization; the control of glucose stimulated insulin secretion; and the mediation of stress responses [71]. It has also been implicated in cardiovascular function, where it is suggested to play a protective role in preventing cardiac hypertrophy [72]. Several recent studies have suggested that there is a link between these two metabolic networks. For example, Hong *et al.* (2014) showed that acetylation of a region needed to maintain the activity of p70 ribosomal S6 kinase (S6K1) blocks its mTORC1-dependent Thr-389 phosphorylation [73]. Thr-389 is a key phosphorylation site needed to maintain S6K1 activity. Acetylation of S6K1 is inhibited by SIRT1 and SIRT2, suggesting that sirtuin regulated acetylation of S6K1 is needed for mTORC1-dependent S6K1 activation [73]. Moreover, it was demonstrated by Ghosh and colleagues using both mouse and human cells that SIRT1 interacts with TSC2, a component of the mTOR inhibitory-complex upstream to mTORC1 and regulates mTOR signaling in a TSC2-dependent manner. These results suggested that SIRT1 negatively regulates mTOR signaling potentially through the TSC1/2 complex [31]. Guo *et al.* (2011) reported similar findings in mice with enhanced neuronal SIRT1 expression. This was accompanied by inhibition of mTOR downstream signaling activity (decreased p70S6 kinase (p70S6K) phosphorylation at Thr389). Interestingly, the authors suggest that Sirt1 may act to promote the growth and survival of neurons in the central nervous system via negatively regulating mTOR signaling [74].

## 4. Therapeutic Avenues for Treating Age-Related Disease?

It has been suggested for some time that the mTOR and SIRT1 signaling pathways play a vital role in the onset and progression of aging and age-related diseases. However, some of the findings surrounding these pathways suggest that a number of possible paradoxes are present in their involvement in aging. mTOR, as has been previously mentioned, is a key cellular regulator of anabolic processes and autophagy. The decreased muscle mass evident from aging would suggest that an upregulation of this signaling pathway would therefore be beneficial. Bodine *et al.* (2001) found that activation of the Akt/mTOR pathway could induce muscular hypertrophy and reduced atrophy in disused limbs in both rats and mice [75]. For those suffering from sarcopenia or age-related muscular atrophy, this could provide a significant step in improving quality of life in later years. The decrease in growth hormone (GH) and insulin-like growth factor (IGF) concentrations that occur with age would suggest that administration could counter the progression of osteoporosis, sarcopenia and even CVD [76]. IGF-1 has been shown to up-regulate the Akt/mTOR pathway and, therefore, to increase protein synthesis [77]. However, an attenuated insulin/IGF-1 signaling has been shown to extend lifespan whilst mutations in IGF-1 receptors in humans have been linked to increased longevity. The interaction of GH and IGF signaling and their effect on aging/longevity have been examined in a number of studies using mice. These have demonstrated that a GH deficiency or a deletion of GH receptors can significantly reduce the effect of aging and increase longevity [76]. As IGF-1 mediates the effects of GH, it has been suggested that it is actually the IGF signaling pathway that is in some way responsible for intensifying the aging process [76]. Once again, in mice with low circulating levels of IGF-1 or with a deletion of the IGF-1 receptors, an increase in longevity in females, but not males, has been found [76]. Rapamycin, is now regularly used as an immunosuppressant to help prevent organ rejection after transplants. Recent data have demonstrated that rapamycin is associated with significantly extending lifespan in mice [78]**,** and it is through the inhibition of the mTOR signaling pathway that it is proposed to exert its effect [79]. Of further interest is the fact that the timing of administration of rapamycin appears to influence its impact. Miller *et al.* (2011) found that administration in mice at nine months (equivalent to ~20 years old in a human) provided no additional benefit compared to those given rapamycin at 20 months (equivalent to ~60 years old in a human) [80]. This suggests that there would not necessarily be a need for lifelong supplementation of rapamycin in order to extend health span, should the same benefit be found in humans.

A further substance that has been strongly linked with the possibility of increasing health span is resveratrol, a plant polyphenol found in grape skins and some other food sources, that has been suggested to mimic calorie restriction [81]. Despite some positive evidence suggesting resveratrol can improve glucose and lipid metabolism, the role this polyphenol plays in cancer and cardiovascular disease progression remains to be fully elucidated [82]. Recent findings have suggested that resveratrol can suppress cellular senescence in human cells; however, this was limited by its toxicity, which occurs at higher concentrations [83]. In contrast, a review by Cottart *et al.* (2014) found that it was relatively well tolerated at doses above 0.5 g/day over long periods [82]. It has been suggested that resveratrol inhibits mTOR signaling pathways by increasing the association between mTOR and its inhibitor DEPTOR, thereby limiting the downstream signaling of mTOR [84]. In addition, an increased expression of SIRT1 has also been postulated as another mechanism by which resveratrol can exert its effect [85]. An increase in SIRT1 could help to reduce oxidative stress, thereby reducing cell damage and ultimately slowing down the aging process. Whilst there have been some positive findings with regards to supplementation strategies that may help to slow down the aging process, there is still some way to go before these methods can be definitively said to improve/limit the process of aging. In summary, both rapamycin and resveratrol have shown some positive effects in various animal models; however, there is a much stronger argument for rapamycin being a candidate for extending health span (reviewed in [36]). However, there is still some debate as to the level of impact possible and if the positive effects observed in a mouse model will truly transfer to the human level [82].

## 5. Mathematical Approaches to Modelling of Biological Pathways

The exact mechanisms that underpin the interaction between SIRT1 and mTOR remain to be discovered. Unravelling their relationship is hampered by the complexity of the pathways that they regulate. Therefore, a full understanding of their interconnectivity is difficult to envisage in the near future. Consequently, we propose breaking the interactions into well-characterized regulatory circuits with mTOR and SIRT1 hubs within the circuits, then using computational modelling in conjunction with experimentation to gain a deeper mechanistic understanding of their interactivity. To do this, it is important to appreciate how to construct a mathematical model of a biological pathway, as several approaches can be employed. Here, we briefly discuss some of the approaches that have commonly been used in systems biology and give examples of where they have been applied to aging studies. We also briefly highlight the advantages and disadvantages of using these approaches.

**Figure 2:**
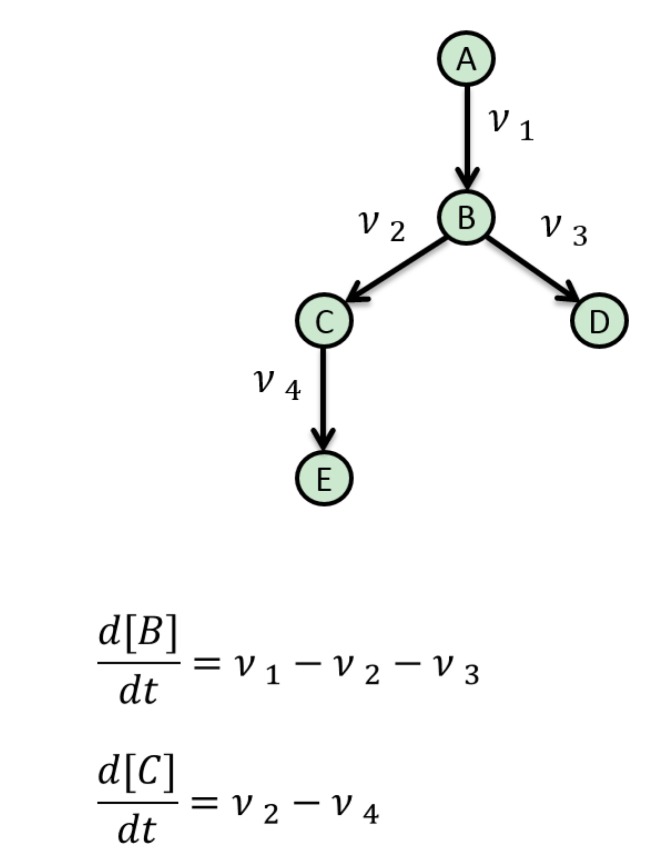
A simple pathway illustrating a metabolic flux model.

One important method that has been successfully applied to metabolic networks is flux balance analysis (FBA). In this approach, each intermediate metabolite pool is assumed to be in a steady state. This defines a set of mass balance constraints that must be satisfied by any feasible flux pattern and that can be solved efficiently for large, even genome-scale models, to understand global interactions [86]. Figure 2 provides a simple pathway to illustrate the concept. Metabolite B, for example, is produced by flux $\nu {\;}_{1}$ and consumed by fluxes $\nu {\;}_{2}$ and $\nu {\;}_{3}$. If the concentration of B is assumed not to vary over time, its rate of change (first derivative with respect to time) can be set to zero, which implies the following simple relationship between these fluxes: $\nu {\;}_{1}-\nu {\;}_{2}-\nu {\;}_{3}=0$. A similar analysis of Metabolite C gives another constraint: $\nu {\;}_{2}-\nu {\;}_{4}=0$. For larger networks, the balancing of hundreds or thousands of metabolites gives the same number of constraints that the fluxes must satisfy. This “constraint space” can be efficiently searched by mathematical optimization algorithms to find a flux pattern that optimizes a certain objective, such as maximization of the cell growth rate. FBA has been widely used to interpret metabolic data, for example in a study on the effect of aging on key metabolite fluxes in hypoxia tolerance in Drosophila [87]. Although originally devised for steady-state metabolic networks, these methods have found some limited application for dynamic signaling networks of the type considered here [88] and also gene regulation [89]. From an aging perspective, several other models are worth mentioning; for example, the FBA model of mitochondrial energy metabolism by Ramakrishna and colleagues [90]. This model was used to characterize the optimal flux distributions for maximal ATP production in the mitochondrion. The model predicted the expected ATP yields for glucose, lactate and palmitate alongside the secretion of TCA-cycle intermediates, which is observed during mitochondrial disease [90]. More recently, Nogiec and colleagues (2015) used FBA modelling to create a systems-level model of insulin resistance, under a variety of nutrient conditions. The metabolic network was probed to isolate reactions that replicate the clinical manifestations of an insulin resistance-linked metabolic state [91]. Yizhak *et al.* (2013) also focused on the area of healthy ageing and generated a metabolic transformation algorithm [92]. This algorithm has the goal of identifying health states within a metabolic network that has been perturbed by disease. According to the authors, the algorithm was able to predict novel drug targets for human ageing based on analyses of several genes associated with lifespan extension in yeast. In terms of disadvantages, flux models are not based on mechanistic biochemical kinetics; therefore, they are limited at predicting metabolic conditions. This means that although the “transportation infrastructure” of the cell is quite well characterized by FBA, the kinetic rates, information exchange and regulation that control the flow of molecules around these metabolic networks are far less well understood. They are also solely based on model steady states, and fluxes are inferred based on steady states. This is not a true reflection of reality, as regulatory processes are inherently dynamic in nature and, therefore, require an alternative dynamic or time-dependent modelling approach [93].

The most ubiquitous approach for dynamic modelling of biological pathways involves the second class of computational models discussed here, namely dynamic deterministic simulations using ordinary differential equations (ODEs). In these models, the state variables of the system can be metabolites or anything in which the concentration is dynamically altered over time by reactions that produce or consume them. These reactions can be enzyme-catalysed transformations of metabolites, post-translational modifications of proteins or synthesis reactions, such as transcription and translation. Unlike FBA models, ODE models require some mechanistic understanding of the reactions involved, as well as estimates of the associated kinetic parameters. This requirement for parameter values limits the size of ODE models, even more than computational tractability. The typical ODE model size is a few reactions or a few tens of reactions for larger models, whereas FBA models frequently approach the so-called “genome scale” of a few hundred or several thousand reactions. The goal of deterministic ODE models is to encapsulate the dynamics of the system by capturing the behaviour of reaction velocities. Several recent excellent ODE models have been applied to the understanding of aging. For example, a recent mathematical model by Kowald *et al.* (2012) was used in conjunction with experimental analysis of Podospora anserina strains in which the mitochondrial superoxide dismutase, PaSOD3, was enhanced [94]. This combined approach was able to indicate that superoxide may not be the primary ROS responsible for age-related molecular decline, which has significant ramifications for the free radical theory of aging [6,8]. Another worthwhile recent model by Proctor *et al.* (2014) utilized an ODE approach and stochastic methods (see below) to assess the therapeutic intervention points for the prevention of age-related cytokine-induced cartilage breakdown. The model predicted that simulated inhibition of c-Jun *N*-terminal kinase or p38 mitogen-activated protein kinases and overexpression of tissue inhibitor of metalloproteinases 3 (TIMP-3) led to a reduction in collagen release [95]. One of the disadvantages associated with using this approach is parameter uncertainty. This presents a challenge for developing reliable ODE models of biological pathways. Some recent methods to address this issue are discussed in Section 6 and Section 7. Further disadvantages include the fact that ODE models are often based on the assumption of spatial homogeneity, and thus, the challenge of incorporating widely different timescales needs to be overcome. This implicitly assumes that a considerable number of molecules take part in the reactions and that the average behaviour of the population of molecules is not affected by individual heterogeneity. This assumption may not be valid for certain signaling proteins and transcripts that have low copy numbers per cell, and this may require the use of stochastic models. Therefore, the final approach that will be discussed briefly is stochastic modelling. This type of model is used if there is variability within the biological system [96]. A stochastic simulator can be utilized if a small number of molecules are suggested to be involved in discrete random collisions within a biochemical/molecular pathway. Computationally, this approach involves an algorithm treating each reaction in the model as a probability function, e.g., biochemical reactions have different probabilities of taking place, which can be altered depending on the nature of the reaction [97,98,99]. There is now considerable experimental evidence to support the application of this framework to a broad range of biological processes [100,101]. It is recognized that stochasticity contributes to aging [102]; thus, it is important to consider this for any future model of mTOR and SIRT1 interplay. Recently stochastic models have been used to represent cellular oxidative damage [103], telomere shortening [104], heat shock protein homeostasis [105] and a variety of biochemical pathways involved in the aging process. The disadvantages of stochastic modelling mainly centre on the fact that, in the main computational systems biology, researchers tend to rely heavily on the Gillespie algorithm or one of its variants (a statistically exact method). Although simple to use and intuitive to understand, this method is extremely computationally intensive, as it must simulate every single reaction [98,99,106].

## 6. Resources for Model Assembly

Assembling a model of a metabolic network or a signaling pathway using the reaction-based approach requires a number of things. Firstly, it is important to establish if the model has been assembled before. This may involve consulting a database, such a BioModels [107]**,** that archives models that have been encoded in the model exchange format systems biology mark-up language (SBML) [108]. It also requires detailed knowledge of the metabolic reactions and their patterns of interaction. This reaction list can be converted into to a network diagram that explicitly outlines the nature of the reactions. This is more formal that a biological diagram of the system and aims to represent the network in a very precise manner. There are a number of ways of creating a network diagram. Recently, systems biology graphical notation has been suggested as a standardized method for generating network diagrams [109]. Reaction modelling also requires detailed knowledge of each reaction and their mechanisms. This often depends on the availability of kinetic data, which can be obtained from databases, such as the BRaunschweig ENzyme DAtabase (BRENDA) [110], the System for the Analysis of Biochemical Pathways - Reaction Kinetics (SABIO-RK) [111] or BioNumbers [112]. If kinetic data are available, but the values are different, as they have been obtained using a wide variety of experimental conditions, then it is necessary to build this uncertainty into the model. Uncertain kinetic values can be considered in the modelling process using a sensitivity analysis, which investigates the relationship between uncertain model inputs and the resulting variation in the model outputs. Information on the reactions can also be derived from wet-laboratory observations of time-course behaviour. These data are necessary in order to infer a reasonable parameter set to establish the validity of the model. This is important, as the utility of the model is dependent to a large extent on how complete the knowledge is of the metabolic system. A software tool is also necessary for model building. It is now unnecessary to have an advanced understanding of mathematics and computer programming, as significant progress has been made recently and there is a plethora of freely available modelling tools that are intuitive and reasonably straightforward to use (reviewed in [113]). Many software tools are capable of running simulations of the metabolic network model and also have built-in features that allow modellers to conduct sensitivity analysis or parameter estimation.

## 7. Current Limitations and Future Developments

**Figure 3:**
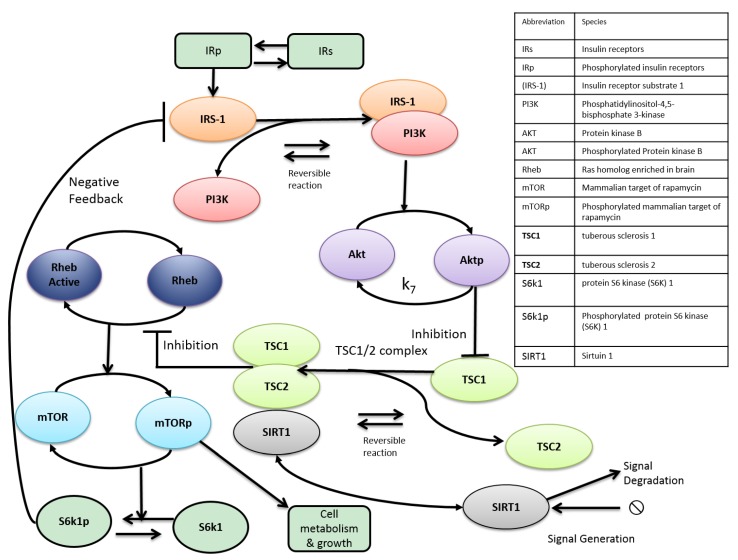
Proposed reduced model of SIRT1 and mTOR interaction based on Ghosh *et al.* (2010) [31]. Insulin binds to IRs resulting in autophosphorylation. IRs can be phosphorylated or unphosphorylated [123]. The active IR induces the phosphorylation of IRS-1 via the activity of protein tyrosine kinase. This signal depends on phosphorylated IR. IRS1p docks with PI3-kinase. This results in the formation of the IRS-1/activated PI3-kinase complex (IRS1pPI3). Dissociation of the complex is dependent on IRS1pPI3. Phosphorylation of Akt depends on IRS1pPI3 and is reversible. Formation of the TSC1/2 complex depends on phosphorylated Akt. The TS1/2 complex inhibits the conversion of inactive Rheb to active Rheb (Rheb-GTP). RhebA activates mTORC1. Activation of mTORC1 affects S6K, while the activity of S6K is triggered by phosphorylation [124]. The activity of S6 kinase triggers feedback mechanisms inhibiting IRS-1. mTORp provokes cell metabolism and growth. We explore the proposed interaction between mTOR and SIRT1 by having SIRT1 bind with the TSC1/2 complex to inhibit active Rheb formation. Thus, the SIRT1 TSC1/2 complex association is included as an inhibitor of active Rheb formation and subsequent mTOR signaling, as suggested by Ghosh and colleagues.

There a number of issues that can impede the development of a model in this area. Firstly, a large number of biological systems remain poorly understood mechanistically. This makes an integrated mathematical description of their mechanisms inherently challenging. Moreover, this problem feeds forward into parameter selection; if the mechanisms have not been fully delineated, then it is probable that there will be a paucity of measurements with which to infer accurate parameter values. A variety of techniques have been developed for estimating parameters that are not identifiable in the strict mathematical sense [114,115]. Residual parameter uncertainty, however, has an impact on model extension, because if the parameters of the original model are unclear, this raises questions over the value of extending the model further. This is particularly frustrating from the perspective of the aging process, as due to its intrinsic complexity, holistic models are a necessity if we are to improve our understanding of this process. To help overcome these difficulties, Kriete *et al.* (2010) created a noteworthy rule-based cell systems model of aging that included the mTOR pathway along with several other pathways related to aging [116]. This semi-quantitative model enabled the authors to represent aging in a holistic fashion that was defined by connectivity/rules among all components involved. Another promising direction for future research is the development of algorithmic methods for reducing model complexity and identifying, from the ensemble of uncertain model parameters, the critical components that demand further investigation. A good example of this is sensitivity analysis to determine those parameters that exert the most influence over a particular model output [117]. The performance of formal techniques for automated model reduction of systems biology models has also been studied by Dokoumetzidis and Aarons [118]. Recently, Rao and colleagues (2014) developed a model reduction method for biochemical reaction networks [119]. The method employed a stepwise reduction in the number of complexes, defined as the left- and right-hand sides of the reactions in the network. The reduced network has fewer complexes, reactions, variables and parameters when compared to the original network, and yet, the behaviour of a preselected set of significant metabolites in the reduced network closely resembles that of the original network. Moreover, the reduced network largely retains the structure and kinetics of the original model. A yeast model of glycolysis was reduced from 12 to seven variables. This reduction was able to improve the understanding of the dynamics of the network and presents a means of facilitating model parameterization or to embed a detailed model of interest in a more coarse-grained, yet realistic environment [120]. This type of approach could be important for modelling the interaction between mTOR and SIRT1, because their metabolic activities comprise a multitude of relationships that encapsulates the dynamic behaviour between enzymes, metabolites and cofactors. Given the current limited understanding of the mechanisms that underpin these pathways and the cloudiness surrounding their interactivity, constructing a detailed model of their circuitry is to a large extent futile. Rather, it is vital that computational models are informed by recent experimental evidence and that they are used to test a particular idea or hypothesis relating to a component or components of a reasonably well-defined system. The incorporation of model entities needs to be informed by metabolic circuitry that has been at least partly characterized experimentally. As an example, we created a diagram that could be used as a starting point to model the interaction of mTOR and SIRT1 (Figure 3). This diagram is informed by the hypothesis outlined by Ghosh *et al.* (2010) [31], in which the authors suggest SIRT1 negatively regulates mTOR signaling through inhibition of the TSC1/2 complex. Our idea centres on capturing this effect together with the key components of this pathway. The next step would be to code our putative model using a software tool, such as CellDesigner [121] or Copasi [122], both of which have been designed to support the SBML exchange framework, which would facilitate model updating. It would then be necessary to parameterize the model. Following this, simulations could be conducted along with a sensitivity analysis. The model could then be ultimately used to explore the relationship between mTOR and SIRT1 based on current experimental knowledge.

## 8. Conclusions

Western populations are gradually aging. With this demographic shift comes a concomitant increase in the prevalence of age related-disease. Thus, there is an urgent need to better understand the mechanisms that underpin aging. In this article, two metabolic pathways have been proposed as fundamental to the progress of aging and age-related disease. The importance of these pathways is emphasized by their effects on a diverse range of cellular and metabolic processes. Both pathways are modulated by CR, and recent evidence suggests that there is significant crosstalk between these two metabolic hubs. However, the precise mechanisms that underpin their interactions remain unclear. One way to establish a more complete understanding of the interactions between mTOR and SIRT1 is to use mechanistic computational modelling. There are a number of approaches that can be utilized to describe the proposed mechanistic interplay between these two metabolic circuits. In general, computational models are based on reactions and utilize ordinary differential equations, with the concentrations of metabolic entities and parameters inferred from continuous deterministic data to replicate the behaviour of the systems. The major limitation of this approach is that it depends on an adequate parameter set being inferred from the experimental data. Presently, this is a challenge, as a full kinetic description of these pathways has yet to be established. In this paper, we propose that computational modelling focus on the experimentally well-understood region within the complex circuitry of these metabolic networks. Therefore, we propose that a well-characterized experimental region of a larger circuit is modelled initially to gain a firm understanding of its mechanisms. This is a process that is cyclical in nature and involves hypothesis generation followed by the gathering of experimental evidence to gradually unravel the mechanisms that underpin the metabolic circuitry. Once delineated, these pathways may lead to new therapeutic avenues that could increase health span or even delay aging.
